# Cardiovascular and mortality events in type 2 diabetes cardiovascular outcomes trials: a systematic review with trend analysis

**DOI:** 10.1007/s00592-018-1253-5

**Published:** 2018-11-19

**Authors:** Lorenzo M. Vetrone, Francesco Zaccardi, David R. Webb, Sam Seidu, Nitin N. Gholap, Dario Pitocco, Melanie J. Davies, Kamlesh Khunti

**Affiliations:** 1 Diabetes Research Centre, Leicester Diabetes Centre, Leicester General Hospital, Gwendolen Rd, Leicester, LE5 4PW England UK; 2 Servizio di Diabetologia, Catholic University School of Medicine, Largo Francesco Vito 1, 00198 Rome, Italy; 3 University Hospital of Coventry and Warwickshire, Coventry, Clifford Bridge Rd, Coventry, CV2 2DX England UK

**Keywords:** Cardiovascular, Type 2 diabetes, Randomised trials, Mortality, Trend, Systematic review

## Abstract

**Aims:**

To investigate cardiovascular disease and mortality trends in control arm participants of diabetes cardiovascular outcome trials (CVOTs).

**Methods:**

We electronically searched CVOTs published before October 2017. Data on all-cause mortality, cardiovascular mortality and events, and baseline characteristics were collected, along with study calendar years. Trends were estimated using negative binomial regressions and reported as rate ratio (RR) per 5-year intervals.

**Results:**

26 CVOTs, conducted from 1961 to 2015, included 86788 participants with 6543 all-cause deaths, 3265 cardiovascular deaths, and 7657 3-point major adverse cardiovascular events (3-P MACE; combined endpoint of cardiovascular death, nonfatal myocardial infarction, nonfatal stroke). In unadjusted analysis, there was an increasing trend for 3-P MACE rates over time (5-year RR 1.57; 95% CI 1.34, 1.84); a small increasing trend for cardiovascular disease mortality rates (1.13; 1.01, 1.26); and stable rates for all-cause death. Adjusting for age, sex, previous myocardial infarction, and diabetes duration, there was no evidence of trends for 3-P MACE or cardiovascular disease mortality rates, while reducing rates were observed for nonfatal myocardial infarction (5-year RR: 0.72; 0.54, 0.96), total stroke (0.76; 0.66, 0.88), and nonfatal stroke (0.60; 0.43, 0.82).

**Conclusions:**

In contrast to real-world data, there was no evidence of an improvement in all-cause and cardiovascular mortality in type 2 diabetes participants included in control arms of randomised clinical trials across 5 decades. Further studies should investigate whether and how dissimilarities in populations, procedures, and assessments of exposures and outcomes explain the differences between real-world setting and clinical trials.

**Electronic supplementary material:**

The online version of this article (10.1007/s00592-018-1253-5) contains supplementary material, which is available to authorized users.

## Introduction

Type 2 diabetes mellitus (T2DM) is a complex cardiometabolic disorder affecting approximately 8.5% of the global population [1]. Subjects with T2DM have 2–3-times higher risk of cardiovascular disease and death and [2–4], at 40 years of age, have an estimated 8 years shorter life expectancy than subjects without diabetes [3].

Elevated plasma glucose concentration is consistently and directly associated with cardiovascular complications and mortality in multiple, large epidemiological studies in people with T2DM; yet, whether treatment of hyperglycaemia and in particular intensive glucose control translates into a lower risk of cardiovascular disease and mortality remains uncertain. Along with the possible benefit of glucose reduction, a better “control” of other cardiovascular risk factors such as dyslipidaemia and hypertension and an earlier identification of diabetes through screening have likely contributed to the declining rates of diabetes-related complications and mortality in the last two decades [5], as shown in large observational studies from Sweden [6], US [7], and Australia [8].

In contrast with real-life settings, most participants of RCTs have a single or few medical conditions, are younger, and are possibly more adherent to medications (Hawthorne effect) [9]. To assure high internal validity and reduce the variation in baseline risk factors, RCTs use strict inclusion criteria and commonly exclude very ill patients. These factors may potentially contribute to differences between real-world and RCTs in terms of both treatment effects and absolute risk of disease-related outcomes. However, evidence from RCTs is likewise relevant as it complements observations from other sources and it is considerably less prone to bias arising from outcomes definition and assessment, incomplete data collection, and observational confounding [10]. In contrast to “real-world” evidence, a systematic assessment of trends of diabetes-related outcomes from randomised controlled trials (RCTs) is lacking [11–13]. Indeed, recent systematic evaluations included only RCTs published up to March 2011 [13], while the number of available RCTs reporting cardiovascular outcomes has increased significantly since 2008, when the US Food and Drug Administration (FDA) mandated inclusion of cardiovascular outcomes trials (CVOTs) in safety assessments of newer glucose-lowering drugs [14].

In this view, we aimed to systematically investigate trends over the last five decades in cardiovascular events and mortality rates in T2DM patients enrolled in the control arm of RCTs assessing effectiveness of various interventions, including glucose-lowering therapies. We estimated trends in the control arm as the management of glucose and other risk factors in these participants had to follow the best-standard-of-care at the time when RCTs were conducted; therefore, the risk of cardiovascular and all-cause death in control arm participants is closer to the “real-world” risk, particularly if the treatment is associated with important absolute effects.

## Materials and methods

### Data sources and searches

This study was conducted according to a pre-specified protocol and followed standard guidelines for conducting and reporting systematic reviews (PRISMA checklist reported in the Supplemental Material) [15]. We searched PubMed and the Cochrane Central Register of Controlled Trials (CENTRAL) for RCTs published in English from inception until 21 October 2017.

### Study selection

Following the PICOS (population, intervention, comparator, outcome, study design) framework, we included RCTs (study design) of any duration in adult patients with T2DM (population) randomised to a specific treatment or strategy (intervention and comparators) and reporting cardiovascular outcomes or mortality (outcome); details on the search strategy are reported in the Supplemental Material. Reference lists of retrieved articles were also manually scanned for all relevant additional studies and reviews. Studies were included if: (1) outcome-specific or mortality number of events and person-years were reported; (2) it was possible to calculate them from the mean/median follow-up, rates, or the total number of participants. When multiple observational follow-ups of the same RCTs were available, we included only the main study (shorter duration) to have a more precise estimate of the rates related to the specific calendar year (Supplemental Table S1).

### Data extraction and quality assessment

We used standardised, pre-defined forms for data extraction and quality assessment. Three authors extracted the data independently on: first author name; RCT acronym; year of journal publication; ClinicalTrials.gov (NCT) and PubMed ID (PMID) identifier number; follow-up duration; RCT calendar years (start and end); randomisation treatments; population type and baseline characteristics; mortality and cardiovascular outcomes data. Study quality was assessed using the Cochrane risk of bias tool and disagreement at any stage was solved by consensus or arbitration [16].

### Data synthesis and analysis

For each study and outcome, we extracted the number of events (*Ev*) and exposure time (person-years, *PY*) in the control (placebo) arm. If *Ev* or *PY* were not reported, they were estimated using the following formulae: *Ev* = rate × *PY* and *PY* = mean (or median) follow-up × number of participants. We estimated trends using negative binomial regression with $Ev$ as numerator, $PY$ as denominator, and calendar time (defined as the mid-point between start and end of the RCT) as continuous variable; trends of incident rates [with 95% confidence intervals (CI)] were displayed in forest plots as rate ratio per 5-year intervals (i.e., ratio comparing 5-year increments of calendar time). To account for possible study-level differences, outcome-specific regressions were progressively adjusted for baseline age, sex, prevalence of myocardial infarction, and diabetes duration.

We performed three sensitivity analyses. First, we estimated adjusted trends excluding studies with patients recruited before year 2000 (mid-point); this post-hoc analysis was decided at the writing stage of the discussion paragraph of the manuscript, to facilitate a temporal comparison of our findings with trends reported in observational studies. Second, at revision stage, we estimated adjusted rate ratios including baseline HbA1c. Lastly, three RCTs (HEART 2D, TOSCA and DEVOTE; references of studies are reported in the Supplemental Material) were included in the main analysis although there were no control arms: for these studies, we pre-planned to use *Ev* and *PY* of all participants and to assess the consistency of these results with those obtained after their exclusion.

We used STATA v. 15.0 (Stata Corp, College Station, TX, USA) for data manipulation, analyses, and graphs; *p* value < 0.05 was considered statistically significant.

## Results

### Study characteristics

After duplicates exclusion and selection of articles by title and abstract, 46 reports underwent full-text assessment and 26 were included in the quantitative analysis (Supplemental Figure S1); reasons for exclusion of the remaining 20 studies are reported in Table S1: for some studies, it was not possible to estimate person-years of follow-up while others did not include T2DM patients; five studies reported longer observational follow-up after the main RCT.

The characteristics of the included RCTs are shown in Table 1: they span 5 decades, from 1966 to 2015, and enrolled a total of 86,788 (median 2656; range 80–8212) participants with T2DM; most studies (21/26, 80.7%) were conducted after year 2000. Baseline age, HbA1c, and disease duration weighted means were 61.7 years, 7.8% (62.3 mmol/mol), and 9.9 years, respectively, and 63.7% were males. Four RCTs included only subjects with prevalent myocardial infarction while a single RCT only patients without; in the remaining RCTs, the prevalence of myocardial infarction ranged from 2 to 52.7% (with higher prevalence in more recent RCTs); follow-up ranged from 1.5 to 10 years.

**Table 1:** Characteristics of included randomised controlled trials

Randomised Controlled Trial (RCT)	PubMed ID	No. of participants	Male (%)	Follow-up (years)^a^	Age (years)^a^	HbA1c (%)^a^	HbA1c (mmol/mol)^a^	Diabetes duration (years)^a^	Prevalent myocardial infarction (%)	RCT calendar year (start–end)	RCT type^b^
UGDP	4926376	205	30.7	7.0	55.1	–	–	0	3.0	1961–1966	SD
UKPDS 33	9742976	1138	62.0	10.0	53.4	7.1	54.1	0	2.0	1977–1991	SD
DCGP^c^	23549519	620	53.1	5.8	65.4	10.2	88.0	0	7.7	1989–1991	SD
STENO-2	12556541	80	70.0	7.8	55.2	8.8	72.7	6.0	2.5	1993–1993	SD
JDCS	20054522	1016	53.0	7.8	58.6	7.9	62.8	10.9	0	1995–1996	SD
VADT	19092145	899	97.1	5.6	60.3	9.4	79.2	11.5	19.0	2000–2003	SD
PROactive	16214598	2633	66.0	2.9	61.6	7.9	62.8	8.0	46.1	2001–2002	PS
LOOK AHEAD	23796131	2575	40.3	9.6	58.9	7.3	56.3	5.0	6.1	2001–2003	SD
RECORD	19501900	2227	51.7	5.5	58.5	7.9	62.8	7.1	5.1	2001–2003	PS
ADVANCE	18539916	5569	57.7	5.0	66.0	7.5	58.5	8.0	12.0	2001–2003	SD
ACCORD	18539917	5123	61.6	3.5	62.2	8.3	67.2	10.0	18.1	2001–2005	SD
ADDITION	21705063	1379	57.3	5.3	60.2	7.0	53.0	0	6.1	2001–2006	SD
HEART2D	19246588	1115	63.3	2.7	61.0	8.3	67.2	9.1	100	2002–2005	SD
ORIGIN^d^	22686416	6273	66.8	6.2	63.5	6.4	46.4	5.3	35.2	2003–2005	PS
TECOS	26052984	7339	70.5	3.0	65.5	7.2	55.2	11.6	42.5	2008–2012	PS
TOSCA	28917544	3028	58.5	4.8	62.3	7.7	60.6	8.4	6.4	2008–2014	PS
EXAMINE	23992602	2679	68.0	1.5	61.0	8.0	63.9	7.3	100	2009–2013	PS
CANVAS	28605608	4347	63.3	3.6	63.4	8.2	66.1	13.7	50.8	2009–2015	PS
SAVOR-TIMI	23992601	8212	66.6	2.0	65.0	8.0	63.9	10.3	37.6	2010–2011	PS
LEADER	27295427	4672	64.0	3.8	64.4	8.7	71.6	12.9	30.0	2010–2012	PS
ALECARDIO	24682069	3610	72.5	2.0	61.0	7.8	61.7	8.6	100	2010–2012	PS
ELIXA	26630143	3034	69.1	2.1	60.6	7.6	59.6	9.4	100	2010–2013	PS
EMPAREG	26378978	2333	72.0	2.9	63.2	8.1	65.0	9.5	46.4	2010–2013	PS
EXSCEL	28910237	7396	62.0	3.2	62.0	8.0	63.9	12.0	52.7	2010–2015	PS
SUSTAIN-6	27633186	1649	60.0	2.1	64.6	8.7	71.6	13.6	32.9	2013–2013	PS
DEVOTE	28605603	7637	62.6	2.0	50.0	8.4	68.3	16.4	34.1	2013–2014	PS

Trials are listed by starting calendar year (older to newer) and number of participants (largest to smallest); their references are reported in the supplementary material. Data shown for control arm (except DEVOTE, TOSCA, HEART2D where arms were combined)

^a^Mean/median; ^b^*SD* strategy-driven study, *PS* product-specific study; ^c^563 total participants for total myocardial infarction outcome and 591 for total stroke; ^d^in ORIGIN, 11.15% of the population had impaired glucose tolerance or impaired fasting glucose at baseline

– Not available

RCTs reported several outcomes (Table 2 and Table S2): data were complete for all-cause mortality (26 RCTs, 86,788 participants, 6543 events), followed by cardiovascular disease mortality (19 RCTs, 71,405 participants, 3265 events), total stroke (i.e., any type; 16 RCTs, 53,157 participants, 1948 events), and 3-point major adverse cardiovascular events (3-P MACE definitions are reported in Table S3; 15 RCTs, 71,641 participants, 7657 events).

**Table 2:** Number of events and rates of included randomised controlled trials

Randomised controlled trial (RCT)	All-cause mortality		Cardiovascular disease mortality		Major adverse cardiovascular events^a^		Total myocardial infarction		Nonfatal myocardial infarction		Total stroke		Nonfatal stroke	
	*n*	Rate	*n*	Rate	*n*	Rate	*n*	Rate	*n*	Rate	*n*	Rate	*n*	Rate
UGDP	21	14.6	10	7.0	–	–	–	–	–	–	–	–	–	–
UKPDS 33	213	18.9	–	–	–	–	186	17.4	101	9.5	55	5.0	44	4.0
DCGP	147	48.0	–	–	–	–	75	27.3	–	–	50	17.4	–	–
STENO-2	15	24.0	7	11.2	–	–	–	–	17	27.2	–	–	20	32.1
JDCS	43	5.4	–	–	–	–	–	–	–	–	75	9.5	–	–
VADT	95	18.9	29	5.8	–	–	78	15.5	–	–	36	7.2	–	–
PROactive	186	24.5	–	–	–	–	–	–	144	19.0	107	14.1	–	–
LOOK AHEAD	202	8.6	57	2.4	283	12.5	191	8.4	183	8.0	80	3.4	–	–
RECORD	157	12.8	71	5.8	165	13.5	56	4.6	–	–	63	5.1	–	–
ADVANCE	533	19.1	–	–	590	21.2	337	12.1	–	–	246	8.8	–	–
ACCORD	203	11.4	94	5.6	371	22.9	–	–	235	14.5	–	–	61	3.7
ADDITION	92	12.5	–	–	–	–	–	–	–	–	–	–	–	–
HEART2D	102	33.9	86	28.6	–	–	126	41.9	103	34.2	37	12.3	35	11.6
ORIGIN	965	26.0	576	15.5	1013	28.5	326	9.0	–	–	319	8.8	–	–
TECOS	537	24.5	366	16.7	746	36.2	316	15.1	–	–	183	8.7	–	–
TOSCA	105	7.5	–	–	–	–	–	–	45	3.0	–	–	40	2.5
EXAMINE	173	43.1	130	32.4	316	78.6	–	–	–	–	–	–	–	–
CANVAS	307	19.5	201	12.8	496	31.5	198	12.6	183	11.6	151	9.6	132	8.4
SAVOR-TIMI	378	21.0	260	14.5	609	36.0	278	17.0	–	–	–	–	–	–
LEADER	447	25.0	278	16.0	694	39.0	339	19.0	317	18.0	199	11.0	177	10.0
ALECARDIO	138	19.1	98	13.6	360	49.9	–	–	239	33.1	–	–	50	6.9
ELIXA	223	33.0	158	24.0	–	–	261	41.0	–	–	60	9.0	–	–
EMPAREG	194	28.6	137	20.2	282	43.9	–	–	–	–	69	10.5	60	9.1
EXSCEL	584	23.0	383	15.0	905	40.0	493	21.0	–	–	218	9.0	–	–
SUSTAIN-6	60	17.6	46	13.5	146	44.0	–	–	64	19.2	–	–	44	13.1
DEVOTE	423	28.0	278	18.4	681	45.0	–	–	313	23.7	–	–	150	10.7

Trials are listed by starting calendar year (older to newer) and number of participants (largest to smallest); their references are reported in the supplementary material. Data shown for control arm (except DEVOTE, TOSCA, HEART2D where arms were combined)

*n* number of events; rate are per 1000 person-years

^a^3-Point major adverse cardiovascular events: cardiovascular death, nonfatal myocardial infarction, nonfatal stroke (details are reported in Supplementary Table S3)

The overall risk of bias was considered low. For all items and RCTs, it was low, high, and unclear in 86.3%, 7.7%, and 6.0% of the cases, respectively (Table S4). The highest domain-specific bias was observed for “blinding of participants and researchers” (nine RCTs, 34.6%), followed by “binding of outcome assessment” (four RCTs, 15.4%) and “incomplete outcome data” (one RCT, 3.8%).

### Trends

**Fig. 1:**
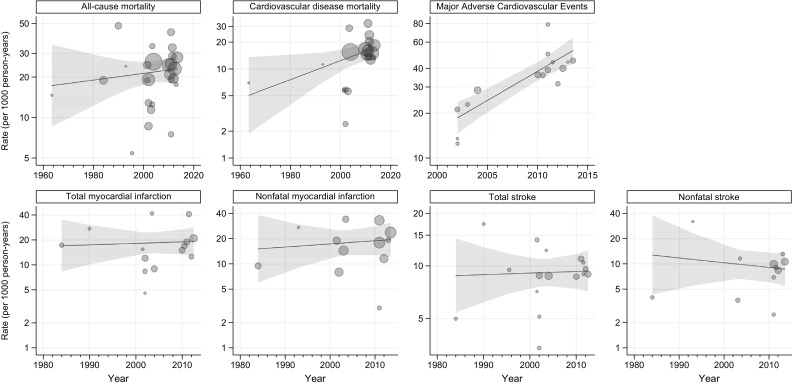
Unadjusted outcome rates by calendar year. Each circle indicates a randomised controlled trial and its size is proportional to the inverse of rate variance. Shadow areas indicate 95% confidence interval

**Fig. 2:**
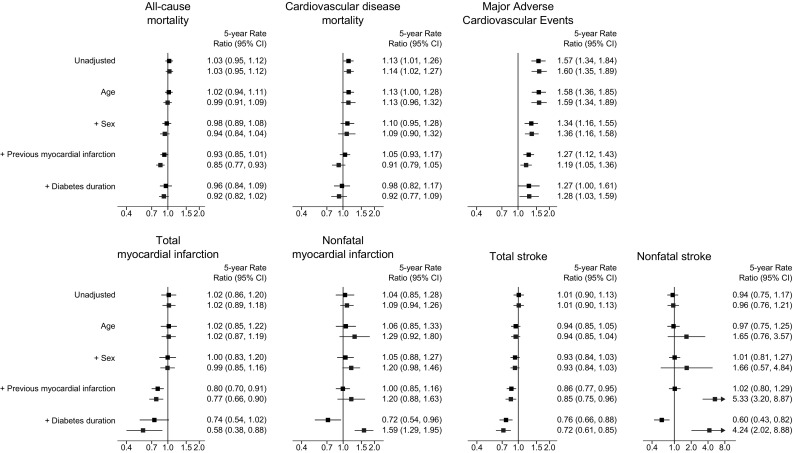
Outcome-specific rate ratios. Black and blue estimates indicate 5-year rate ratios for main and sensitivity analysis (excluding DEVOTE, TOSCA, HEART 2D), respectively. Major adverse cardiovascular events definitions are reported in Supplemental Table S3

In view of the number of events, participants, and outcomes reported (Table S2), we estimated formal trends for all-cause and cardiovascular disease mortality, total and nonfatal stroke, total and nonfatal myocardial infarction, and 3-P MACE. Unadjusted temporal trends are depicted in Fig. 1 and 5-year rate ratios are shown in Fig. 2. With the exception of increasing trend in 3-P MACE (unadjusted 5-year rate ratio 1.57; 95% CI 1.34, 1.84), there was no clear trend for other outcomes while a small increase for cardiovascular disease mortality (unadjusted 5-year rate ratio 1.13; 1.01, 1.26) was observed (Fig. 2). These estimates translated into around 6 and 45 more unadjusted cardiovascular disease deaths and 3-P MACE, respectively, per 1000 person-years comparing 2015 to 2000 (Table S5). All-cause mortality, cardiovascular disease mortality, and 3-P MACE rates were positively related to the baseline prevalence of myocardial infarction and males in included participants, while the relationship with HbA1c (Figure S2) was less clear. Accounting for baseline participants’ characteristics, in the fully adjusted models there was no evidence of trends for all-cause mortality (adjusted 5-year rate ratio 0.96; 95% CI 0.84, 1.09), cardiovascular death (0.98; 0.82, 1.17), or 3-P MACE (1.27; 1.00, 1.61) while reducing rates were observed for nonfatal myocardial infarction (0.72; 95% CI 0.54, 0.96), total stroke (0.76; 0.66, 0.88), and nonfatal stroke (0.60; 0.43, 0.82) (Fig. 2). Rates were conversely rising for cardiovascular disease mortality when the analysis was limited to studies with consistent 3-P MACE definitions (adjusted 5-year rate ratio 1.76; 95% CI 1.13, 2.73; Table S6).

### Sensitivity analyses

Adjusted rate ratios for trends in various outcomes did not materially change when the analysis: was restricted to studies conducted after year 2000, with the exception of nonfatal stroke for which the rate reduction was not significant (adjusted 5-year rate ratio 0.97; 95% CI 0.71, 1.31) (Table S7); or accounted for baseline HbA1c (Table S8). For most outcomes, the results were also consistent excluding HEART 2D, TOSCA and DEVOTE; conversely, there were increasing trends for nonfatal myocardial infarction (adjusted 5-year rate ratio 1.59; 95% CI 1.29, 1.95) and nonfatal stroke (4.24; 2.02, 8.88) (Fig. 2).

## Discussion

In this study, we systematically searched RCTs reporting mortality and cardiovascular events in patients with T2DM randomised to a specific glucose-lowering strategy or to a specific drug, to quantify the rates of these outcomes in control arms and describe their trends. As RCTs included participants from mid-1960 to 2015, it was possible to quantify outcome trends across 5 decades. We found no important changes over the observed years in most of the relevant diabetes-related outcomes, including death from cardiovascular causes, myocardial infarction, or death from any cause; 3-P MACE, a combined cardiovascular endpoint commonly used in RCTs (particularly recent ones), showed an increasing trend which, however, was less evident when accounting for participants’ baseline characteristics across RCTs. Notable exceptions were the declining trends for the individual outcome total stroke, nonfatal stroke, and nonfatal myocardial infarction, and a possible reduction for total myocardial infarction.

Recent decades have been characterised by significant improvements in the diagnosis and treatment of cardiovascular disease risk factors. As a result, declining trends of major cardiovascular disease have been repeatedly reported in observational studies from several countries, both in the general population and in people with T2DM [7] (Table S9). As cardiovascular diseases represent the main cause of death in patients with T2DM, such reduction also translates in a lower mortality risk [2], albeit with wide variation in absolute mortality rates across different countries. The reasons behind such heterogeneity in mortality rates are likely related to clinical (including access to healthcare; screening, early detection and management of T2DM and its complications; proactive ongoing management of hyperglycaemia and other risk factors; patient education and self-management; and prevalent comorbidities), biological/genetic, and socioeconomic differences. Along with the multifaceted syndemic interplay between these elements [17], differences in the processes of measuring (data quality, exposure definitions and assessment, outcome ascertainment) and synthesising (study design and analysis) information could also have contributed. Such heterogeneity in mortality rates was also observed in RCTs included in our analysis; however, in this situation it is more likely attributable to clinical differences rather than study design and analysis.

Variations in rates of single and combined cardiovascular outcomes comparing observational studies and RCTs are more difficult to interpret than mortality. Differences in the definitions and ascertainment of outcomes are well recognised in observational studies (i.e., physician vs self-reported T2DM or cardiovascular outcome), particularly for fatal events, where there are spatiotemporal differences in the definition and reporting of the underlying cause of death [18, 19]. In an attempt to limit heterogeneous comparisons, efforts have been made to standardise definitions of cardiovascular outcomes and their composites in RCTs, thus making geographical and temporal comparisons more reliable. With this in mind, our results indicate a nonsignificant 30% increased risk of major adverse cardiovascular events every 5 years, accounting for differences in demographic and clinical characteristics of RCTs’ participants. These results are possibly linked to rising trends of cardiovascular mortality seen in the analysis of RCTs reporting 3-P MACE, while the contribution of nonfatal myocardial infarction and nonfatal stroke to this trend is uncertain. In fact, there are only seven studies with stratified data for these two outcomes among the RCTs reporting 3-P MACE.

When including all available RCTs, however, we found declining trends for both nonfatal myocardial infarction and stroke. The divergent trends between cardiovascular death and nonfatal cardiovascular events have several possible explanations. More intensive glucose control in recent years (change in glycemic targets), coupled with an increased prevalence of diabetes in multimorbid elderly patients, may have resulted in increasing rates of hypoglycaemia which has been associated with a higher risk of cardiovascular death in post-hoc analysis of RCTs, observational, and experimental studies [20–23]. There is also a possibility that other cardiovascular phenotypes are increasingly contributing to the risk of cardiovascular death. The reduction of cardiovascular death attributable to fatal atherothrombosis due to a widespread use of statin and aspirin, along with the increased risk of heart failure associated with aging [24], could have changed the phenotype “responsible” for the majority of cardiovascular complications and cardiovascular deaths in patients with T2DM, with a shift from myocardial infarction and stroke to chronic heart failure [25–27]. The recent suggestion to include heart failure in CVOTs as a pre-specified component of MACE would help in reducing the misclassification of outcomes and clarify whether and how changes in the cardiovascular death phenotype explain the contrasting trends between fatal and nonfatal events observed in this analysis [24]. Further insights will also be provided by several ongoing CVOTs which included only T2DM with heart failure or were specifically designed to assess the risk of heart failure [14, 24].

Notably, in the sensitivity analysis excluding HEART 2D, TOSCA and DEVOTE, there was an inversion of trends with rising rates for nonfatal myocardial infarction and nonfatal stroke. These findings are likely related to the very low rates for both outcomes reported in TOSCA (3 per 1000 person-years for nonfatal myocardial infarction and 2.5 for nonfatal stroke) when compared to those observed in other RCTs. The reasons for such a striking difference are unknown although, as pointed out by the investigators of this study, they could be attributable to the ubiquitous use of statins, anti-hypertensive and antiplatelet agents [28].

To our knowledge, this is the first study to assess trends of key diabetes-related outcomes including all CVOTs studies conducted after the 2008 FDA guidance on CVOTs. This resulted in a much larger number of studies compared to previous systematic investigations and therefore in a substantial increase in the statistical reliability of the findings [11–13]. We also extracted data simultaneously on several outcomes and baseline characteristics of included participants, to give as clear a picture as possible of cardiovascular complications adjusted for potential confounders associated with outcomes’ rates. The study has also some limitations. We had no access to patient-level data which would have allowed a more detailed assessment of the contribution of confounders (including cardioprotective drugs, such as β-receptor antagonists, ACE-inhibitors, aldosterone antagonists, statins, and anti-hypertensive treatments) on trends and of a possible presence of ecological (aggregation) bias [29]. Moreover, we were not able to extract information across all studies for other potential study-level confounders; however, we adjusted for key covariates strongly related to the risk of cardiovascular disease and death, namely age, sex, duration of diabetes and, more importantly, prevalent cardiovascular disease [3]. Among other possible cardiovascular diseases at baseline, we selected myocardial infarction because it was the only confounder reported in all studies. The adjustment for prevalent myocardial infarction lessens the impact on the estimates of the different baseline risk of outcomes, particularly when comparing RCTs published after vs before 2008.

In contrast to observational data, in this study there was no evidence from RCTs of reducing rates of all-cause and cardiovascular mortality in patients with T2DM. For both RCTs and observational studies, more homogenous definitions of exposure and outcomes, the inclusion of heart failure among pre-specified endpoints, and an easier access to individual participant data will help quantify the differences between experimental and real-world evidence and further elucidate the reasons behind such divergences. Moreover, as prediction models for cardiovascular disease and mortality risk are instrumental in defining treatment strategies, targets, and clinical guidelines, health care decisions should consider that models’ performance could be highly influenced by the nature of the data, as the absolute risk of events is highly heterogeneous comparing RCTs and “real-world” patients.

## Electronic supplementary material

Below is the link to the electronic supplementary material.

Supplementary material 1 (PDF 897 KB)
