# Supplementary material for: Cardiovascular and mortality events in type 2 diabetes cardiovascular outcomes trials: a systematic review with trend analysis
Source: Acta Diabetol. 2018 Nov 19;56(3):331–9. doi: 10.1007/s00592-018-1253-5 (PMC6394717; doi:10.1007/s00592-018-1253-5)
Supplement: Supplementary file 1 — Supplementary material 1 (PDF 897 KB) [file 592_2018_1253_MOESM1_ESM.pdf]

Supplemental Material

Cardiovascular and mortality events in type 2 diabetes cardiovascular outcomes trials: A systematic review with trend analysis

Lorenzo M Vetrone[a b *], Francesco Zaccardi[a *], David R Webb[a], Sam Seidu[a], Nitin N Gholap[c], Dario Pitocco[b], Melanie J Davies[a], Kamlesh Khunti[a]

* Equally contributed

[a] Diabetes Research Centre, Leicester Diabetes Centre, Leicester General Hospital Gwendolen Rd, Leicester, LE5 4PW, England, UK

[b] Servizio di Diabetologia, Catholic University School of Medicine, Largo Francesco Vito 1, 00198 Roma

[c] University Hospital of Coventry and Warwickshire, Coventry, Clifford Bridge Rd, Coventry, CV2 2DX, England, UK

Contents

Table S1: Studies excluded, with reasons 2

Table S2: Total number of events and participants, by outcome 2

Table S3: Major Adverse Cardiovascular Events (MACE) definitions 3

Table S4: Risk of bias for the included studies 4

Table S5: Rate differences 5

Table S6: Five-year rate ratios for studies with consistent MACE definitions 5

Table S7: Five-year rate ratios excluding studies conducted before year 2000 5

Table S8: Five-year rate ratios adjusted for baseline HbA1c 6

Table S9: Standardised mortality rates in people with diabetes in selected observational studies 7

Figure S1: PRISMA flow chart and search strategy 8

Figure S2: Study-level baseline characteristics and outcome rates 9

References of the included randomised controlled trials 10

Table S1: Studies excluded, with reasons

Randomised Controlled Trial	PubMed ID	Reason for exclusion
SDIS	3048052	Type 1 diabetes
KUMAMOTO-6	7587918	Mean follow-up and rates unavailable
KUMAMOTO-8	10860187	Mean follow-up and rates unavailable
Wing et al	14527106	Non-randomized study
DIGAMI 2	15728645	Inpatient treatment
HOME	19307526	Mean follow-up and rates unavailable
Finnish DPS	19479072	Patients with Impaired Glucose Tolerance only
BARI 2D	19502645	No randomization to glucose-lowering/intensive glucose treatment
NAVIGATOR	20228402	Patients with Impaired Glucose Tolerance only
ACCORD follow-up	20427682	Secondary epidemiological analysis
J-EDIT	22435936	Only composite data available
PROFIT-J	24477028	Only composite data available
Da Qing	24731674	Patients with Impaired Glucose Tolerance only
ACE	28917545	Patients with Impaired Glucose Tolerance only
PROactive follow-up	26592506	Follow-up study
UKPDS follow-up	18784090	Follow-up study
STENO-2 2008 follow-up	18256393	Follow-up study
STENO-2 2016 follow-up	27531506	Follow-up study
DCGP follow-up	23549519	Follow-up study
UKPDS 34	9742977	Subgroup of the main UKPDS study

Table S2: Total number of events and participants, by outcome

	Number of			
Outcome	Randomised Controlled Trials	Events	Participants	Person-years
All-cause mortality	26	6543	86788	322074
Cardiovascular disease mortality	19	3265	71405	241661
Total stroke	16	1948	53157	229621
*Major Adverse Cardiovascular Events	15	7657	71641	244273
Total myocardial infarction	14	3260	55359	221236
Nonfatal myocardial infarction	12	1944	37607	133017
Nonfatal stroke	11	813	34732	111785
Fatal myocardial infarction	5	113	9705	46696
Silent myocardial infarction	3	97	8120	24788
Fatal stroke	3	45	6925	39549
Total myocardial infarction (no silent)	1	126	2333	6528
Nonfatal myocardial infarction (no silent)	1	121	2333	6541
Total ischemic stroke	1	141	8212	16588

Outcomes are listed by number of randomised controlled trials.
* MACE: definitions reported in Table S3.

Table S3: Major Adverse Cardiovascular Events (MACE) definitions

Randomised Controlled Trial	Major Adverse Cardiovascular Events (MACE) definition
ACCORD	Nonfatal myocardial infarction, nonfatal stroke, death from cardiovascular causes
ADVANCE	Cardiovascular death, nonfatal myocardial infarction, nonfatal stroke
ALECARDIO	Cardiovascular mortality, nonfatal myocardial infarction, nonfatal stroke
CANVAS	Death from cardiovascular causes, nonfatal myocardial infarction, nonfatal stroke
DEVOTE	Death from cardiovascular causes, nonfatal myocardial infarction, nonfatal stroke
EMPAREG	Death from cardiovascular causes, nonfatal myocardial infarction (excluding silent myocardial infarction), nonfatal stroke
EXAMINE	Death from cardiovascular causes, nonfatal myocardial infarction, nonfatal stroke
EXSCEL	Death from cardiovascular causes, nonfatal myocardial infarction, nonfatal stroke
LEADER	First occurrence of death from cardiovascular causes, nonfatal (including silent) myocardial infarction, nonfatal stroke
LOOK AHEAD	Death from cardiovascular causes, nonfatal myocardial infarction, nonfatal stroke
ORIGIN	Death from cardiovascular causes, nonfatal myocardial infarction, nonfatal stroke
RECORD	Cardiovascular death, myocardial infarction, stroke
SAVOR-TIMI 53	Cardiovascular death, myocardial infarction, stroke
SUSTAIN-6	Death from cardiovascular causes, nonfatal myocardial infarction (including silent), nonfatal stroke
TECOS	Cardiovascular death, nonfatal myocardial infarction, nonfatal stroke

Trials are listed by acronym alphabetical order.

Table S4: Risk of bias for the included studies

Randomised Controlled Trial	Random Sequence	Allocation Concealment	Blinding of participants and researchers	Blinding of outcome assessment	Incomplete outcome data	Selective reporting data	Other
ACCORD	Low	Unclear	High	Low	Low	Low	Low
ADDITION	Low	Low	High	Low	Low	Low	Low
ADVANCE	Low	Low	Low	Low	Low	Low	Low
ALECARDIO	Low	Low	Low	Low	Low	Low	Low
CANVAS	Low	Low	Low	Low	High	Low	Low
DCGP	Low	Low	High	High	Low	Low	Unclear
DEVOTE	Low	Low	Low	Low	Low	Low	Low
ELIXA	Low	Low	Low	Low	Low	Low	Low
EMPAREG	Low	Low	Low	Low	Low	Low	Low
EXAMINE	Low	Low	Low	Low	Low	Low	Low
EXSCEL	Low	Low	Low	Low	Low	Low	Low
HEART2D	Low	Low	High	High	Low	Low	Low
JDCS	Low	Low	High	High	Unclear	Low	Unclear
LEADER	Low	Low	Low	Low	Low	Low	Low
LOOK AHEAD	Low	Low	High	Low	Low	Low	Low
ORIGIN	Low	Low	High	Low	Low	Low	Unclear
PROactive	Low	Low	Low	Low	Low	Low	Low
RECORD	Low	Low	Low	Low	Low	Low	Low
SAVOR-TIMI 53	Low	Low	Low	Low	Low	Low	Low
STENO-2	Low	Low	High	Low	Low	Low	Low
SUSTAIN-6	Low	Low	Low	Low	Low	Low	Unclear
TECOS	Low	Low	Low	Low	Low	Low	Low
TOSCA	Low	Low	High	High	Low	Low	Low
UGDP	Low	Low	Unclear	Unclear	Low	Low	Unclear
UKPDS 33	Low	Low	Low	Low	Low	Low	Unclear
VADT	Low	Low	Low	Low	Low	Low	Unclear
**OVERALL**	**100% Low**	**96.2% Low**	**61.5% Low**	**80.8% Low**	**92.3% Low**	**100% Low**	**73.1% Low**
	**0% Unclear**	**3.8% Unclear**	**3.9% Unclear**	**3.8% Unclear**	**3.9% Unclear**	**0% Unclear**	**26.9% Unclear**
	**0% High**	**0% High**	**34.6% High**	**15.4% High**	**3.8% High**	**0% High**	**0% High**

Trials are listed by acronym alphabetical order.

Table S5: Rate differences

Outcome	Comparison (years)	Rate difference per 1000 person-years (95% CI)
All-cause mortality	2015 vs 2000	1.9 (-3.8, 7.7)
Cardiovascular disease mortality	2015 vs 2000	5.5 (0.2, 10.8)
Major Adverse Cardiovascular Events*	2015 vs 2000	44.7 (27.3, 62.0)
Total myocardial infarction	2010 vs 2000	0.7 (-5.4, 6.9)
Nonfatal myocardial infarction	2010 vs 2000	1.5 (-5.9, 8.9)
Total stroke	2010 vs 2000	0.2 (-1.9, 2.3)
Nonfatal stroke	2010 vs 2000	-1.3 (-5.7, 3.2)

Estimates reported for unadjusted models in the main analysis.
* 3-point Major Adverse Cardiovascular Events: cardiovascular death, nonfatal myocardial infarction, nonfatal stroke. Detailed are reported in Table S3.
For myocardial infarction and stroke, the comparison was 2010 vs 2000 in view of studies availability over time (Figure 1). Other comparison years have been selected to facilitate the comparison with calendar time reported in observational studies available in the literature (Table S9).

Table S6: Five-year rate ratios for studies with consistent MACE definitions

Outcome	Analysis	No. of RCTs	5-year Rate Ratio	P-value
Cardiovascular disease mortality	Main	14	1.76 (1.13, 2.73)	0.012
	Sensitivity	13	1.65 (1.04, 2.62)	0.035
Nonfatal myocardial infarction	Main	7	1.92 (1.52, 2.43)	<0.001
	Sensitivity	6	0.35 (0.10, 1.15)	0.083
Nonfatal stroke	Main	7	2.26 (1.48, 3.46)	<0.001
	Sensitivity	6	1.42 (0.65, 3.08)	0.376

Estimates adjusted for age, sex, previous myocardial infarction and diabetes duration. Sensitivity analyses excluded HEART 2D, TOSCA and DEVOTE trial.

Table S7: Five-year rate ratios excluding studies conducted before year 2000

Outcome	Analysis	No. of RCTs	5-year Rate Ratio	P-value
All-cause mortality	Main	21	0.98 (0.82, 1.17)	0.861
	Sensitivity	18	1.02 (0.85, 1.23)	0.840
Cardiovascular disease mortality	Main	17	1.28 (0.96, 1.71)	0.087
	Sensitivity	15	1.64 (1.05, 2.56)	0.028
Major Adverse Cardiovascular Events	Main	15	1.27 (1.00, 1.61)	0.046
	Sensitivity	14	1.28 (1.03, 1.59)	0.023
Total myocardial infarction	Main	12	0.90 (0.68, 1.19)	0.473
	Sensitivity	11	0.92 (0.52, 1.60)	0.760
Nonfatal myocardial infarction	Main	10	0.72 (0.53, 0.98)	0.038
	Sensitivity	7	1.46 (1.11, 1.92)	0.006
Total stroke	Main	13	0.86 (0.72, 1.04)	0.131
	Sensitivity	12	0.82 (0.64, 1.05)	0.120
Nonfatal stroke	Main	9	0.97 (0.71, 1.31)	0.840
	Sensitivity	6	1.42 (0.65, 3.08)	0.376

Estimates adjusted for age, sex, previous myocardial infarction and diabetes duration. Excluded studies (DGCP; JDCS; STENO-2; UGDP; UKPDS 33) had mid-point calendar year between start and end of recruitment <2000. Sensitivity analyses excluded HEART 2D, TOSCA and DEVOTE trial.

Table S8: Five-year rate ratios adjusted for baseline HbA1c

Outcome	Analysis	No. of RCTs	5-year Rate Ratio	P-value
All-cause mortality	Main	25	1.01 (0.84, 1.20)	0.949
	Sensitivity	22	0.94 (0.80, 1.11)	0.475
Cardiovascular disease mortality	Main	18	1.12 (0.82, 1.52)	0.479
	Sensitivity	16	1.37 (0.81, 2.31)	0.242
Major Adverse Cardiovascular Events	Main	15	1.27 (1.01, 1.60)	0.040
	Sensitivity	14	1.29 (1.08, 1.53)	0.005
Total myocardial infarction	Main	14	0.85 (0.63, 1.15)	0.287
	Sensitivity	13	0.68 (0.46, 1.00)	0.049
Nonfatal myocardial infarction	Main	12	0.89 (0.69, 1.15)	0.370
	Sensitivity	9	0.94 (0.68, 1.30)	0.695
Total stroke	Main	16	0.80 (0.69, 0.92)	0.002
	Sensitivity	15	0.75 (0.65, 0.88)	<0.001
Nonfatal stroke	Main	11	0.90 (0.65, 1.24)	0.513
	Sensitivity	8	4.33 (2.60, 7.21)	<0.001

Estimates adjusted for age, sex, previous myocardial infarction, diabetes duration, and baseline HbA1c. Sensitivity analyses excluded HEART 2D, TOSCA and DEVOTE trial.

Table S9: Standardised mortality rates in people with diabetes in selected observational studies

Country	Study	Age at baseline (mean/median)	Year		Rate (per 1000 PYs)		Note	Reference (PubMed ID)
			Start	End	Start	End		
SWEDEN	Swedish National Diabetes Register (NDR)	65.2	1998	2013	40.6	34.4	T2DM	28402770
SCOTLAND	Scottish Care Information-Diabetes (SCI-Diabetes)	60.0	2004	2013	19.8	18.1	T2DM	27465219
US	National Health Interview Survey Linked Mortality	60.0	1988	2015	23.1	15.2	T1 & T2DM	29784146
US	National Center for Health Statistics survey	60.0	1997	2006	20.3	15.1	T1 & T2DM	22619288
SWEDEN	Three national Swedish registries	62.7	2006	2013	15.5	14.3	T2DM on any medication	27189067
UK	The Health Improvement Network (THIN) database	62.9	1996	2009	31.4	14.1	T1 & T2DM	24114114
CANADA	Ontario diabetes database	60.2	1995	2005	17.6	13.3	T1 & T2DM	17336651
CANADA	Population-based healthcare databases (Ontario)	61.0	1996	2009	19.4	12.2	T1 & T2DM	24114114
AUSTRALIA	National Diabetes Service Scheme (NDSS) registry	58.5	2000	2011	9.7	7.9	T2DM	27208325
AUSTRALIA	National Diabetes Service Scheme (NDSS) registry	60.0	1997	2010	9.4	5.5	T2DM	24947787

Studies are listed by mortality rate at the end of observation time
T1DM: Type 1 diabetes mellitus; T2DM: Type 2 diabetes mellitus;

Selected observational studies reporting standardised mortality rates in people with diabetes in the last two decades.
PubMed Keywords search: diabetes[title] AND (trends[title] OR rates[title] OR trend[title] OR rate[title]) AND (mortality[title] OR death[title]) on 30/05/2018.

PubMed ID	Reference
28402770	Rawshani A et al. *Mortality and Cardiovascular Disease in Type 1 and Type 2 Diabetes*. N Engl J Med. 2017; 376:1407-1418
27465219	Read SH et al. *Trends in type 2 diabetes incidence and mortality in Scotland between 2004 and 2013*. Diabetologia. 2016; 59:2106-13
29784146	Gregg EW et al. *Trends in cause-specific mortality among adults with and without diagnosed diabetes in the USA: an epidemiological analysis of linked national survey and vital statistics data.* Lancet. 2018; 391:2430-2440
22619288	Gregg EW et al. *Trends in death rates among U.S. adults with and without diabetes between 1997 and 2006: findings from the National Health Interview Survey*. Diabetes Care. 2012; 35:1252-7
27189067	Norhammar A et al. *Incidence, prevalence and mortality of type 2 diabetes requiring glucose-lowering treatment, and associated risks of cardiovascular complications: a nationwide study in Sweden, 2006-2013*. Diabetologia. 2016; 59:1692-701
24114114	Lind M et al. *Mortality trends in patients with and without diabetes in Ontario, Canada and the UK from 1996 to 2009: a population-based study*. Diabetologia. 2013; 56:2601-8
17336651	Lipscombe LL et al. *Trends in diabetes prevalence, incidence, and mortality in Ontario, Canada 1995-2005: a population-based study*. Lancet. 2007; 369:750-6
27208325	Harding JL et al. *Age-Specific Trends From 2000-2011 in All-Cause and Cause-Specific Mortality in Type 1 and Type 2 Diabetes: A Cohort Study of More Than One Million People*. Diabetes Care. 2016; 39:1018-26
24947787	Harding JL et al. *Mortality trends among people with type 1 and type 2 diabetes in Australia: 1997-2010*. Diabetes Care. 2014; 37:2579-86

Figure S1: PRISMA flow chart and search strategy

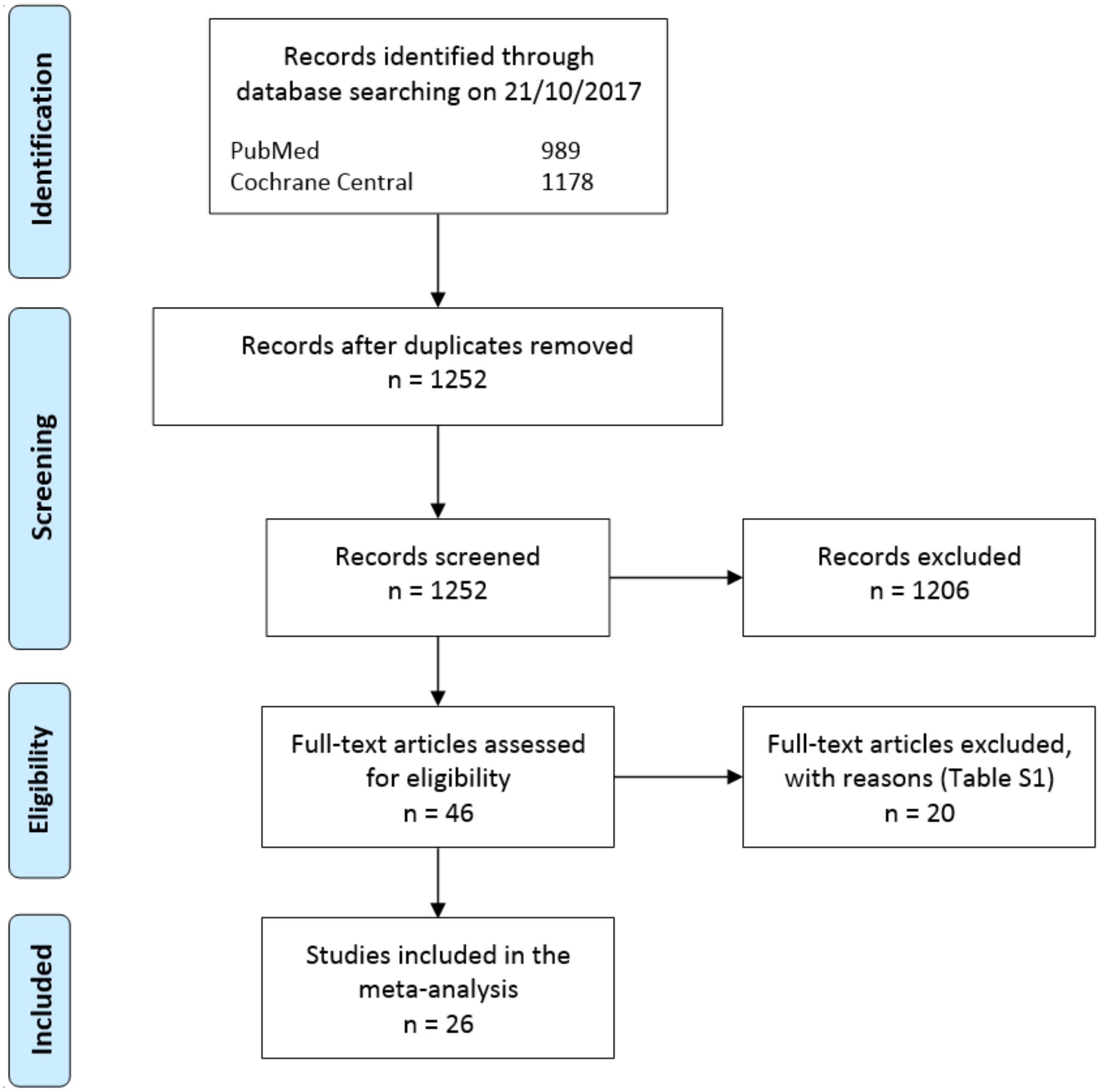

Search Strategy

Articles published in English from inception to 21/10/2017 were searched in PubMed and Cochrane Central databases. Electronic search was supplemented by scanning reference lists of all relevant articles, including reviews, by hand searching of relevant journals.

For PubMed, the search was:
((Diabetes[Title]) *AND* (Fatal[Title/Abstract] *OR* Stroke[Title/Abstract] *OR* Infar*[Title/Abstract] *OR* Death[Title/Abstract] *OR* Mortality[Title/Abstract])) *NOT* Type 1[Title] Filters: Clinical Trial; Humans

For Cochrane Central, the search was:
#1 "diabetes":ti in Trials; **#2** "fatal":ti,ab,kw or "stroke":ti,ab,kw or "infarct":ti,ab,kw or "infarction":ti,ab,kw or "death":ti,ab,kw; **#3** "mortality":ti,ab,kw; **#4** "type 1 diabetes mellitus":ti,ab,kw or "type 1 diabetes" or "type 1 DM" or "type 1 diabetic"
#5 #1 and (#2 or #3) not #4

Figure S2: Study-level baseline characteristics and outcome rates

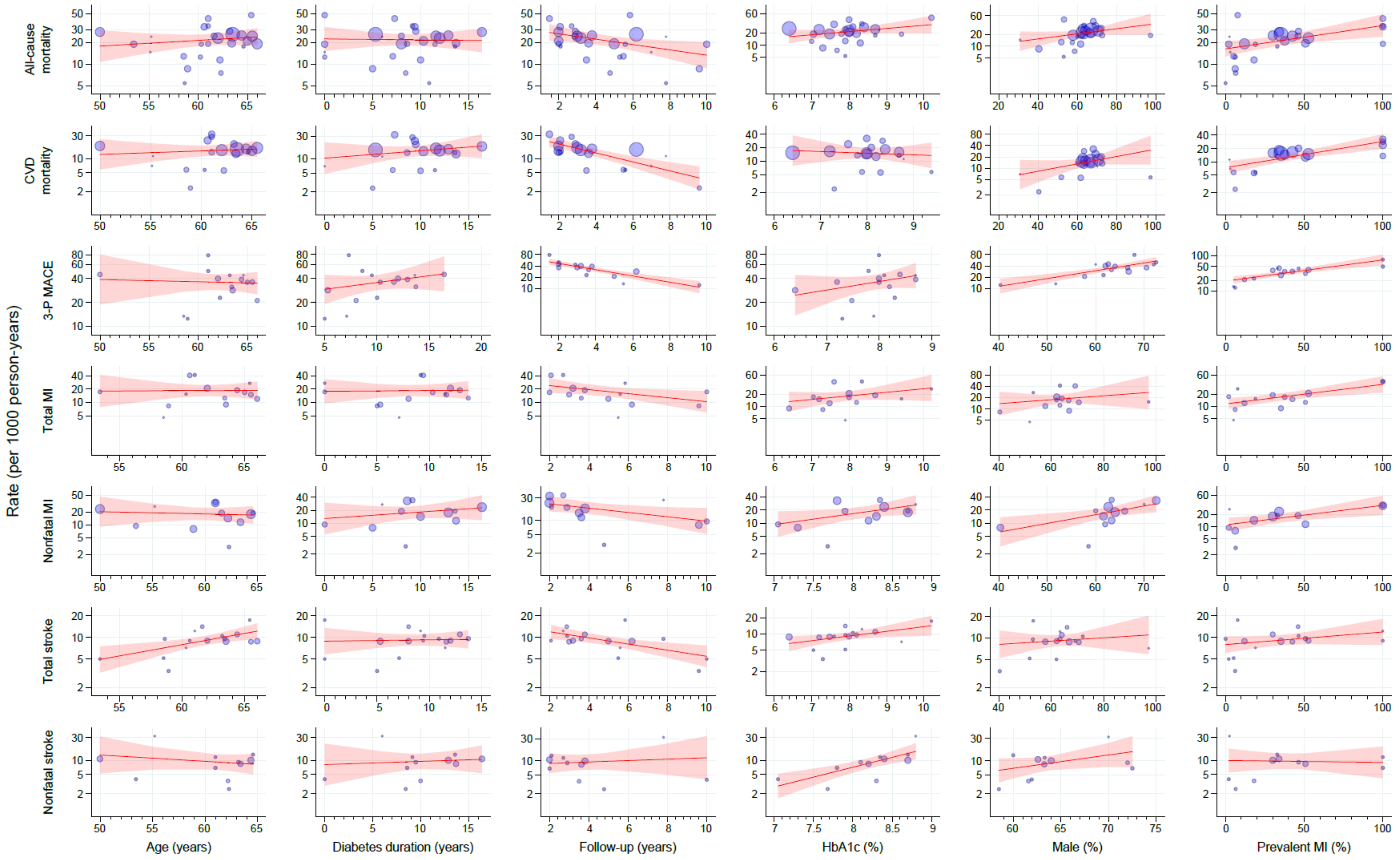

Each circle indicates a randomised controlled trial and its size is proportional to the inverse of rate variance. Shadow areas indicate 95% confidence interval.

References of the included randomised controlled trials

RCT acronym	PubMed ID	Reference
ACCORD	18539917	1
ADDITION	21705063	2
ADVANCE	18539916	3
ALECARDIO	24682069	4
CANVAS	28605608	5
DCGP	23549519	6
DEVOTE	28605603	7
ELIXA	26630143	8
EMPAREG	26378978	9
EXAMINE	23992602	10
EXSCEL	28910237	11
HEART2D	19246588	12
JDCS	20054522	13
LEADER	27295427	14
LOOK AHEAD	23796131	15
ORIGIN	22686416	16
PROactive	16214598	17
RECORD	19501900	18
SAVOR-TIMI	23992601	19
STENO-2	12556541	20
SUSTAIN-6	27633186	21
TECOS	26052984	22
TOSCA	28917544	23
UGDP	4926376	24
UKPDS 33	9742976	25
VADT	19092145	26

Trials are listed by acronym alphabetical order.

1. Action to Control Cardiovascular Risk in Diabetes Study G, Gerstein HC, Miller ME, Byington RP, Goff DC, Jr., Bigger JT, Buse JB, Cushman WC, Genuth S, Ismail-Beigi F, Grimm RH, Jr., Probstfield JL, Simons-Morton DG and Friedewald WT. Effects of intensive glucose lowering in type 2 diabetes. *N Engl J Med*. 2008;358:2545-59.

2. Griffin SJ, Borch-Johnsen K, Davies MJ, Khunti K, Rutten GE, Sandbaek A, Sharp SJ, Simmons RK, van den Donk M, Wareham NJ and Lauritzen T. Effect of early intensive multifactorial therapy on 5-year cardiovascular outcomes in individuals with type 2 diabetes detected by screening (ADDITION-Europe): a cluster-randomised trial. *Lancet*. 2011;378:156-67.

3. Group AC, Patel A, MacMahon S, Chalmers J, Neal B, Billot L, Woodward M, Marre M, Cooper M, Glasziou P, Grobbee D, Hamet P, Harrap S, Heller S, Liu L, Mancia G, Mogensen CE, Pan C, Poulter N, Rodgers A, Williams B, Bompoint S, de Galan BE, Joshi R and Travert F. Intensive blood glucose control and vascular outcomes in patients with type 2 diabetes. *N Engl J Med*. 2008;358:2560-72.

4. Lincoff AM, Tardif JC, Schwartz GG, Nicholls SJ, Ryden L, Neal B, Malmberg K, Wedel H, Buse JB, Henry RR, Weichert A, Cannata R, Svensson A, Volz D and Grobbee DE. Effect of aleglitazar on cardiovascular outcomes after acute coronary syndrome in patients with type 2 diabetes mellitus: the AleCardio randomized clinical trial. *JAMA*. 2014;311:1515-25.

5. Neal B, Perkovic V, Mahaffey KW, de Zeeuw D, Fulcher G, Erondu N, Shaw W, Law G, Desai M, Matthews DR and Group CPC. Canagliflozin and Cardiovascular and Renal Events in Type 2 Diabetes. *N Engl J Med*. 2017;377:644-657.

6. Hansen LJ, Siersma V, Beck-Nielsen H and de Fine Olivarius N. Structured personal care of type 2 diabetes: a 19 year follow-up of the study Diabetes Care in General Practice (DCGP). *Diabetologia*. 2013;56:1243-53.

7. Marso SP, McGuire DK, Zinman B, Poulter NR, Emerson SS, Pieber TR, Pratley RE, Haahr PM, Lange M, Brown-Frandsen K, Moses A, Skibsted S, Kvist K and Buse JB. Efficacy and Safety of Degludec versus Glargine in Type 2 Diabetes. *N Engl J Med*. 2017;377:723-732.

8. Pfeffer MA, Claggett B, Diaz R, Dickstein K, Gerstein HC, Kober LV, Lawson FC, Ping L, Wei X, Lewis EF, Maggioni AP, McMurray JJ, Probstfield JL, Riddle MC, Solomon SD, Tardif JC and Investigators E. Lixisenatide in Patients with Type 2 Diabetes and Acute Coronary Syndrome. *N Engl J Med*. 2015;373:2247-57.

9. Zinman B, Wanner C, Lachin JM, Fitchett D, Bluhmki E, Hantel S, Mattheus M, Devins T, Johansen OE, Woerle HJ, Broedl UC, Inzucchi SE and Investigators E-RO. Empagliflozin, Cardiovascular Outcomes, and Mortality in Type 2 Diabetes. *N Engl J Med*. 2015;373:2117-28.

10. White WB, Cannon CP, Heller SR, Nissen SE, Bergenstal RM, Bakris GL, Perez AT, Fleck PR, Mehta CR, Kupfer S, Wilson C, Cushman WC, Zannad F and Investigators E. Alogliptin after acute coronary syndrome in patients with type 2 diabetes. *N Engl J Med*. 2013;369:1327-35.

11. Holman RR, Bethel MA, Mentz RJ, Thompson VP, Lokhnygina Y, Buse JB, Chan JC, Choi J, Gustavson SM, Iqbal N, Maggioni AP, Marso SP, Ohman P, Pagidipati NJ, Poulter N, Ramachandran A, Zinman B, Hernandez AF and Group ES. Effects of Once-Weekly Exenatide on Cardiovascular Outcomes in Type 2 Diabetes. *N Engl J Med*. 2017;377:1228-1239.

12. Raz I, Wilson PW, Strojek K, Kowalska I, Bozikov V, Gitt AK, Jermendy G, Campaigne BN, Kerr L, Milicevic Z and Jacober SJ. Effects of prandial versus fasting glycemia on cardiovascular outcomes in type 2 diabetes: the HEART2D trial. *Diabetes Care*. 2009;32:381-6.

13. Sone H, Tanaka S, Iimuro S, Tanaka S, Oida K, Yamasaki Y, Oikawa S, Ishibashi S, Katayama S, Yamashita H, Ito H, Yoshimura Y, Ohashi Y, Akanuma Y and Yamada N. Long-term lifestyle intervention lowers the incidence of stroke in Japanese patients with type 2 diabetes: a nationwide multicentre randomised controlled trial (the Japan Diabetes Complications Study). *Diabetologia*. 2010;53:419-28.

14. Marso SP, Daniels GH, Brown-Frandsen K, Kristensen P, Mann JF, Nauck MA, Nissen SE, Pocock S, Poulter NR, Ravn LS, Steinberg WM, Stockner M, Zinman B, Bergenstal RM, Buse JB, Committee LS and Investigators LT. Liraglutide and Cardiovascular Outcomes in Type 2 Diabetes. *N Engl J Med*. 2016;375:311-22.

15. Look ARG, Wing RR, Bolin P, Brancati FL, Bray GA, Clark JM, Coday M, Crow RS, Curtis JM, Egan CM, Espeland MA, Evans M, Foreyt JP, Ghazarian S, Gregg EW, Harrison B, Hazuda HP, Hill JO, Horton ES, Hubbard VS, Jakicic JM, Jeffery RW, Johnson KC, Kahn SE, Kitabchi AE, Knowler WC, Lewis CE, Maschak-Carey BJ, Montez MG, Murillo A, Nathan DM, Patricio J, Peters A, Pi-Sunyer X, Pownall H, Reboussin D, Regensteiner JG, Rickman AD, Ryan DH, Safford M, Wadden TA, Wagenknecht LE, West DS, Williamson DF and Yanovski SZ. Cardiovascular effects of intensive lifestyle intervention in type 2 diabetes. *N Engl J Med*. 2013;369:145-54.

16. Investigators OT, Gerstein HC, Bosch J, Dagenais GR, Diaz R, Jung H, Maggioni AP, Pogue J, Probstfield J, Ramachandran A, Riddle MC, Ryden LE and Yusuf S. Basal insulin and cardiovascular and other outcomes in dysglycemia. *N Engl J Med*. 2012;367:319-28.

17. Dormandy JA, Charbonnel B, Eckland DJ, Erdmann E, Massi-Benedetti M, Moules IK, Skene AM, Tan MH, Lefebvre PJ, Murray GD, Standl E, Wilcox RG, Wilhelmsen L, Betteridge J, Birkeland K, Golay A, Heine RJ, Koranyi L, Laakso M, Mokan M, Norkus A, Pirags V, Podar T, Scheen A, Scherbaum W, Schernthaner G, Schmitz O, Skrha J, Smith U and Taton J. Secondary prevention of macrovascular

events in patients with type 2 diabetes in the PROactive Study (PROspective pioglitAzone Clinical Trial In macroVascular Events): a randomised controlled trial. *Lancet*. 2005;366:1279-89.

18. Home PD, Pocock SJ, Beck-Nielsen H, Curtis PS, Gomis R, Hanefeld M, Jones NP, Komajda M and McMurray JJ. Rosiglitazone evaluated for cardiovascular outcomes in oral agent combination therapy for type 2 diabetes (RECORD): a multicentre, randomised, open-label trial. *Lancet*. 2009;373:2125-35.

19. Scirica BM, Bhatt DL, Braunwald E, Steg PG, Davidson J, Hirshberg B, Ohman P, Frederich R, Wiviott SD, Hoffman EB, Cavender MA, Udell JA, Desai NR, Mosenzon O, McGuire DK, Ray KK, Leiter LA, Raz I, Committee S-TS and Investigators. Saxagliptin and cardiovascular outcomes in patients with type 2 diabetes mellitus. *N Engl J Med*. 2013;369:1317-26.

20. Gæde P, Vedel P, Larsen N, Jensen GVH, Parving H-H and Pedersen O. Multifactorial Intervention and Cardiovascular Disease in Patients with Type 2 Diabetes. *New England Journal of Medicine*. 2003;348:383-393.

21. Marso SP, Bain SC, Consoli A, Eliaschewitz FG, Jodar E, Leiter LA, Lingvay I, Rosenstock J, Seufert J, Warren ML, Woo V, Hansen O, Holst AG, Pettersson J, Vilsboll T and Investigators S-. Semaglutide and Cardiovascular Outcomes in Patients with Type 2 Diabetes. *N Engl J Med*. 2016;375:1834-1844.

22. Green JB, Bethel MA, Armstrong PW, Buse JB, Engel SS, Garg J, Josse R, Kaufman KD, Koglin J, Korn S, Lachin JM, McGuire DK, Pencina MJ, Standl E, Stein PP, Suryawanshi S, Van de Werf F, Peterson ED, Holman RR and Group TS. Effect of Sitagliptin on Cardiovascular Outcomes in Type 2 Diabetes. *N Engl J Med*. 2015;373:232-42.

23. Vaccaro O, Masulli M, Nicolucci A, Bonora E, Del Prato S, Maggioni AP, Rivellese AA, Squatrito S, Giorda CB, Sesti G, Mocarelli P, Lucisano G, Sacco M, Signorini S, Cappellini F, Perriello G, Babini AC, Lapolla A, Gregori G, Giordano C, Corsi L, Buzzetti R, Clemente G, Di Cianni G, Iannarelli R, Cordera R, La Macchia O, Zamboni C, Scaranna C, Boemi M, Iovine C, Lauro D, Leotta S, Dall'Aglio E, Cannarsa E, Tonutti L, Pugliese G, Bossi AC, Anichini R, Dotta F, Di Benedetto A, Citro G, Antenucci D, Ricci L, Giorgino F, Santini C, Gnasso A, De Cosmo S, Zavaroni D, Vedovato M, Consoli A, Calabrese M, di Bartolo P, Fornengo P, Riccardi G, Thiazolidinediones Or Sulfonylureas Cardiovascular Accidents Intervention Trial study g and Italian Diabetes S. Effects on the incidence of cardiovascular events of the addition of pioglitazone versus sulfonylureas in patients with type 2 diabetes inadequately controlled with metformin (TOSCA.IT): a randomised, multicentre trial. *The lancet Diabetes & endocrinology*. 2017;5:887-897.

24. Meinert CL, Knatterud GL, Prout TE and Klimt CR. A study of the effects of hypoglycemic agents on vascular complications in patients with adult-onset diabetes. II. Mortality results. *Diabetes*. 1970;19:Suppl:789-830.

25. Intensive blood-glucose control with sulphonylureas or insulin compared with conventional treatment and risk of complications in patients with type 2 diabetes (UKPDS 33). UK Prospective Diabetes Study (UKPDS) Group. *Lancet*. 1998;352:837-53.

26. Duckworth W, Abraira C, Moritz T, Reda D, Emanuele N, Reaven PD, Zieve FJ, Marks J, Davis SN, Hayward R, Warren SR, Goldman S, McCarren M, Vitek ME, Henderson WG, Huang GD and Investigators V. Glucose control and vascular complications in veterans with type 2 diabetes. *N Engl J Med*. 2009;360:129-39.
